# Structural equation modeling reveals decoupling of ecological and self-perceived outcomes in a garden box social-ecological system

**DOI:** 10.1038/s41598-022-10178-z

**Published:** 2022-04-19

**Authors:** Laura S. Tuominen, Samuli Helle, Heikki Helanterä, Patrik Karell, Lauri Rapeli, Douglas Richmond, Timo Vuorisalo, Jon E. Brommer

**Affiliations:** 1 Department of Biology, University of Turku, 20014 Turku, Finland; 2 Department of Social Research, University of Turku, 20014 Turku, Finland; 3 Ecology and genetics research unit, University of Oulu, 90014 Oulu, Finland; 4 Department of Bioeconomy, Novia University of Applied Sciences, 10600 Ekenäs, Finland; 5 Department of Biology, Lund University, 22362 Lund, Sweden; 6 The Social Science Research Institute, Åbo Akademi, 20500 Turku, Finland

**Keywords:** Evolution, Environmental sciences, Environmental social sciences

## Abstract

It is well known that green urban commons enhance mental and physical well-being and improve local biodiversity. We aim to investigate how these outcomes are related in an urban system and which variables are associated with better outcomes. We model the outcomes of an urban common—box gardening—by applying the Social-Ecological Systems (SES) framework. We expand the SES framework by analyzing it from the perspective of social evolution theory. The system was studied empirically through field inventories and questionnaires and modeled quantitatively by Structural Equation Modeling (SEM). This method offers powerful statistical models of complex social-ecological systems. Our results show that objectively evaluated ecological outcomes and self-perceived outcomes are decoupled: gardening groups that successfully govern the natural resource ecologically do not necessarily report many social, ecological, or individual benefits, and vice versa. Social capital, box location, gardener concerns, and starting year influenced the changes in the outcomes. In addition, the positive association of frequent interactions with higher self-perceived outcomes, and lack of such association with relatedness of group members suggests that reciprocity rather than kin selection explains cooperation. Our findings exemplify the importance of understanding natural resource systems at a very low “grassroot” level.

## Introduction

Ever-increasing natural resource demands emphasize the issue of their sustainable governance^1,2^. Especially shared natural resources, the commons, need well-functioning governance to avoid overuse leading to a resource depletion, the so-called tragedy of commons^3^. Theoretical and empirical studies in social sciences and evolution have shown that under certain conditions shared natural resources can be governed sustainably^4–7^. Sustainability is broadly defined as the patterns and outcomes to promote human needs while maintaining the biophysical conditions for life^1,8^. To achieve sustainability, we need to integrate both the human needs and ecological aspects as well as variables influencing them, hence treating natural resources as social-ecological systems^6,9^. Here, we study an urban common, box gardening, with the aim to predict what aspects of the resource or resource users associate with better ecological and gardener-perceived outcomes. In our study, ecological outcomes are objectively measured, and gardeners self-assess the outcomes related to their needs. Urban commons are collectively owned and governed resources located in an urban space^10,11^. They give an interesting and relevant context for the study of natural resources as they enhance urban biodiversity and urban resilience while also being subject to land use and interest conflicts^12–14^.

In this study, we apply the Social-Ecological Systems (SES) framework^3,15^ to identify critical variables predicting more positive outcomes in urban box gardening^16^. The SES is a comprehensive framework based on extensive empirical research and designed for discipline integration^3,7,17,18^. The core idea of SES is that because sustainable governance of shared resources is a social dilemma involving collective action, solving it depends on many aspects of participants, the resource system in question, and governance arrangements^5,18^. To this end, the SES framework considers eight sub-systems: the interactions (I) between actors (A), governance system (GS), resource system (RS) and resource units (RU) creating outcomes (O) and operating in wider social, economic and political settings (S), and in relation to other ecosystems (ECO)^3^. Using the SES framework to study urban and suburban areas as well as integration of social and biophysical data has been called for^19^. Here, we measure the ecological and self-perceived outcomes and investigate how they depend on variables from actors, governance and resource subsystems.

We expand the SES framework from the point of view of evolutionary explanations of cooperation^4,20^, and include the relatedness between resource users as an additional indicator^21,22^. We study the influence of relatedness on the outcomes together with the stability of groups and repetition of interactions. While similar variables related to reciprocity and trust can be seen as parts of the traditional SES subsystems^5,23^, we treat them separately for two reasons. First, we wish to facilitate cross-talk between SES literature and evolutionary biology. In evolutionary biology, two contrasting explanations offer to solve the problem of sustainable resource use, even when prudent use of the resource results in personal costs, namely direct fitness benefits through relatives (kin selection), and indirect fitness benefits via reciprocal mutually beneficial cooperation^20^. Relatedness between actors, or association of cooperative individuals increases the probability of cooperation evolving and being stable because the benefits are then shared by family members (kin selection), or other cooperative individuals^4,20^. Repetition of interactions and stability of groups are predicted by game theory to increase cooperation, because they allow reciprocity and thus higher benefits to cooperators than non-cooperators even when they are unrelated^5,23^. Repetition of interactions also increases the possibility of social control and efficiency of group functioning and communication^24,25^. Positing the variables separately facilitates the comparison between the evolutionary explanations for cooperation. Second, by treating these variables as a separate subsystem highlights their position as an intermediate between the individuals and the governance system.

We consider urban gardening in 1 m^2^ garden boxes in public land as a social-ecological system. Urban box gardening is an example of the urban green commons, which includes parks and greenery, allotments, community gardens, and many other forms of shared resources in urban context^12,26^. Urban commons are a relevant case for SES studies, as they offer multiple benefits and ecosystem services^12,27^ while facing also several common governance challenges such as inadequate management of the public space, low volunteer participation, or lack of knowledge and institutional support^13,28^. In industrialized countries urban gardeners are not necessarily economically dependent on urban gardens, but still their popularity is growing^27^. Urban gardening is an example of new forms of self-organization, citizenship, awareness and more cooperative land use^14,29^. Urban box gardening offers an interesting comparison to the community gardens and domestic gardens where food security and well-being benefits, socio-economic attributes and governance structures have been extensively studied^14,16,30^. For example, the box gardens are small with no commercial aspect or food security emphasis, which leads to differences in motivations and therefore in system dynamics^16,27^. As the economic outcomes are limited, it is likely that the non-tangible outcomes, such as joy of gardening, connection with nature, healthy lifestyle and social community, play an important role^28–31^. In addition, the social, personal, and ecological benefits have seldom been studied concurrently, especially with regard to factors that contribute to success in achieving these benefits^28,32^. We assume that urban box gardeners vary in succeeding in resource governance, both in ecological and self-perceived outcomes^32,33^, and aim to model these outcomes and predict them by applying the SES framework.

**Figure 1:**
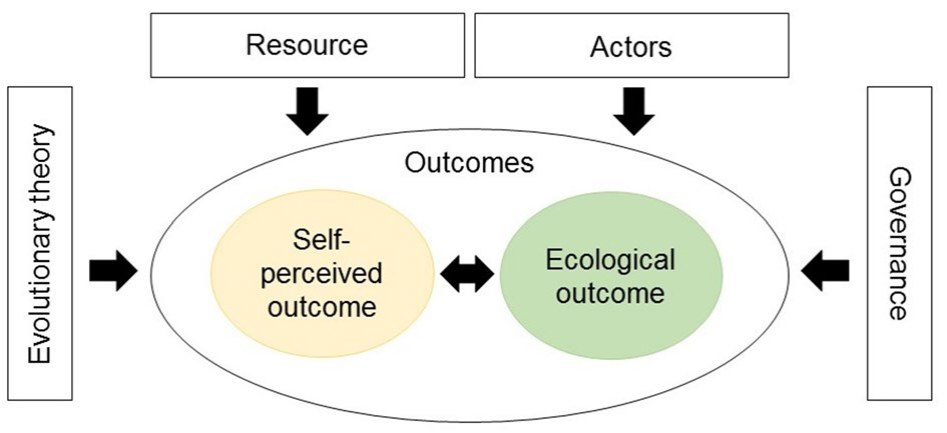
A simplified schematic diagram of the social-ecological system (SES) framework applied for urban gardening system in this study^3^. Sub-systems Actors, Governance, Resource and Evolutionary theory may influence the Outcomes, which itself is composed of Self-perceived and Ecological outcomes. The Self-perceived and Ecological outcomes may or may not be associated with each other in this system. To facilitate dialogue between evolutionary biology and SES literature and combine variables from somewhere between actors and governance, we treat Evolutionary theory as a subsystem.

In the system we focus on, the garden boxes are provided for free by the city to groups of citizens that enroll in the project. Urban garden boxes provide a useful system allowing quantitative analyses of governance^30^. Firstly, as the garden boxes are small and in a public space, an independent observer is able to objectively and precisely quantify ecological outcome, such as diversity, quantity and quality of cultivated species. Secondly, as all the groups are registered when they apply the boxes, it is possible to study gardeners` perceptions and aspects of the social part of the system using an electronic questionnaire. Thirdly, there are several gardening groups governing the garden boxes and each group operates independently, which allows a quantitative comparison of group structure. Typically, in local SES and urban commons case studies, just a few replicate resource systems are compared and studies are more descriptive^7,18,34^, but quantitative modeling of SESs has increased over the recent years after many calls for improvement^19,35^. Modelling multiple outcomes and several putatively important SES-framework subsystems is necessary for analyzing the SESs quantitatively while still accounting for their complexity (Fig. 1).

Our study questions and hypotheses are the following:

1. Is the self-perceived outcome associated with the ecological outcome in the urban garden box system? While outcomes are typically divided into socio-economic and ecological outcomes^18^, here we expect the outcomes to be positively correlated. In this system, the livelihood importance is assumed to be low, but the other benefits are diverse^27^, and therefore, we conceptualize the socio-economic part of the outcomes as the multiple benefits known to be relevant for box gardening. It contains 12 different self-assessed benefits, including benefits related to the cultivations (create biodiversity, self-sufficiency, beautify the area, and fresh vegetables), which are in this case assumed to be positively related with producing good-quality and diverse harvest i.e. ecological outcome^36^. Ecological outcome represents diversely the objectively evaluated aspects of gardening success. In this way, we are also able to investigate if self-assessed ecological outcomes differ from the objectively evaluated ecological outcomes.
2. Which aspects of the SES framework subsystems (actors, governance and resource) are associated with changes in the outcomes in the urban garden box system? Our selection of variables into the study is based on the original framework of Ostrom^2,3^ and prior knowledge on urban gardening^37–39^. We expect a positive association between outcomes and variables related to the resource due to higher resource amount (box number), higher motivation and knowledge (effort), higher productivity (shade), and lower risks outside urban areas (privacy)^36,38,40–42^. We expect the variables from the governance sub-system to have a positive influence on outcomes due to higher motivation and shared strategy (rules), trust in everyone`s participation and shared knowledge (involvement)^2,36,43,44^. Variables related to the actors are expected to have a positive effect on outcomes due to social benefits and aggregate contributions (group size, others number), reciprocity and trust (social capital), and a negative effect due to higher cost of self-organizing (group size), lack of knowledge and experience (starting year), negative experiences (damage) and lack of control on the resource state (worries)^2,3,41,43,44^ (for more details consult Table 1). The variables have not been studied jointly before in this context and therefore we have no clear prediction on how their interrelations will influence the results.
3. What, if any, is the importance of the variables derived from evolutionary theories in the urban garden box system? We expect that stable gardening groups that are composed of relatives, where individuals interact more frequently with each other are more successful in creating positive outcomes due to kin selection, reciprocity and efficiency^4,22^. We examine the variables in a separate subsystem in order to compare the evolutionary explanations for cooperation and increase dialogue between evolutionary biology and SES theory.

**Table 1:** List of Variables Selected for the Study.

SES variable name	Variable name	Operationalization	
**O Outcomes**			
O1 Self-perceived outcome measures	Social benefits	A composite of 4 self-perceived social benefits received from box gardening – stand in the community, quality time with friends or family, community feeling and education of children. For each benefit, the respondent stated whether they had received it; no = 0, a little = 1, a lot = 2. Value 0 (did not receive any of the 4 benefits) – 8 (did receive a lot all of the 4 benefits)	
	Individual benefits	A composite of 4 self-perceived individual benefits received from box gardening – nature connection, know-how, mental relaxation and physical recreation. Value as above	
	Ecological benefits	A composite of 4 self-perceived ecological benefits received from box gardening – create biodiversity, self-sufficiency, beautify the area and fresh vegetables. Value as above	
O2 Ecological outcome measures	Species diversity	Number of cultivated species per box	
	Economic value	Economic value of cultivations per box (€). Economic value of cultivations is equivalent to being bought from a shop × quality × number / area	
	quality	Quality of all cultivations per box (0 dead plant – 5 excellent condition)	
	weeds	Coverage of area of weeds per box (%)	
	number of cultivations	Number of individuals of the cultivated species per box	
	area of cultivations	Area of all cultivations per box	
**Expected effect on O1 and O2**			
**RS Resource system**			
RS3 size of the resource system*	Box number	Gardening box number per group	Positive on O1 & O2 due to more sufficient resource amount^36,40^,however collective action is more challenging when size is very large^3,66^
RS4 human-constructed facilities	effort	Extra effort observed during field inventories (insulation, support built for the plants, boxes painted, weed prevention by garden cover etc., watering system)	Positive on O1 & O2 due to higher motivation and knowledge^32,38^
RS5 productivity of system*	shade	Box location`s exposure to sun < 50% of the day or > 50% of the day	Positive on O1 & O2 due to higher productivity^41^
RS9 location	privacy	Box was located in a more private area (quiet neighborhood, city forest) or less private area (city center, road side)	Positive on O1 & O2 due to lower risks outside the urban center^42^
**Governance system GS**			
GS5 Operational rules*	rules	The respondent stated the group had made a plan for the work beforehand (they had separate boxes or other plan) or the group had not made a plan	Positive on O1 & O2 due to shared rules and customs^2,66^
GS8 Monitoring / sanctioning processes	involvement	The respondent`s view if everyone was involved enough in taking care of the cultivations or not	Positive on O1 & O2 due to trust in more equal participation^43,44^
**Actors A**			
A1 Number of relevant actors*	group size	Number of people in each group	Negative on O2 due to higher costs of self-organizing^3^, positive on O1 due to aggregate contributions^41^
	Others number	Number of people not belonging to the respondent’s group but were box gardening in same location (park / other area)	
A3 history or past experiences	Starting year	The year the respondent had started box gardening (2016 = 0–2019 = 3)	Negative on O1 & O2 due to less experience in solving challenges^41^
	damage	The respondent had experienced some kind of damage done to the boxes during previous years or not	Negative on O1 & O2 due to negative experiences (conflicts)^3,43^
	worries	The respondent`s worries beforehand about vandalism, animal damage or other causes (not at all and just a little = 0 / to some extent and very concerned = 1)	Negative on O1 & O2 due to feeling of inability to control the resource state^44^
A6 norms / social capital*	Social capital	A composite of 8 variables related to social capital—help from others, help towards others, meeting new people, meeting other gardeners, positive or negative feedback from passers-by and a community feeling in the neighborhood or in the gardener`s Facebook group	Positive on O1 & O2 due to norms of reciprocity, trust and positive experiences^2,3,43^
**Evolutionary theory E**			
E1 Repetition of interactions	Group meetings	The frequency of the group meetings during the summer; less than once a month – everyday	Positive on O1 & O2 due to reciprocity and higher efficiency^2,23^
E2 relatedness	relatedness	Respondent`s relatedness to the group members. Their description if people in their gardening group were their family members (children and/or parents), other family members, friends, neighbors or other	Positive on O1 & O2 due to shared benefits between family members^22^
E3 stability of groups	stability	The group composition`s changes over the summer and/or previous or future summers	Positive on O1 & O2 due to reciprocity and trust^23,41^

This table lists the key variables of the Social-Ecological Systems (SES) framework and evolutionary theories for urban gardening system and description of their operationalization in this study. The expected influence on the self-perceived and ecological outcomes are presented for each predicting variable. Variables marked with * are SES variables found to be associated with self-organization (Ostrom 2009, Nagendra & Ostrom 2014).

**Figure 2:**
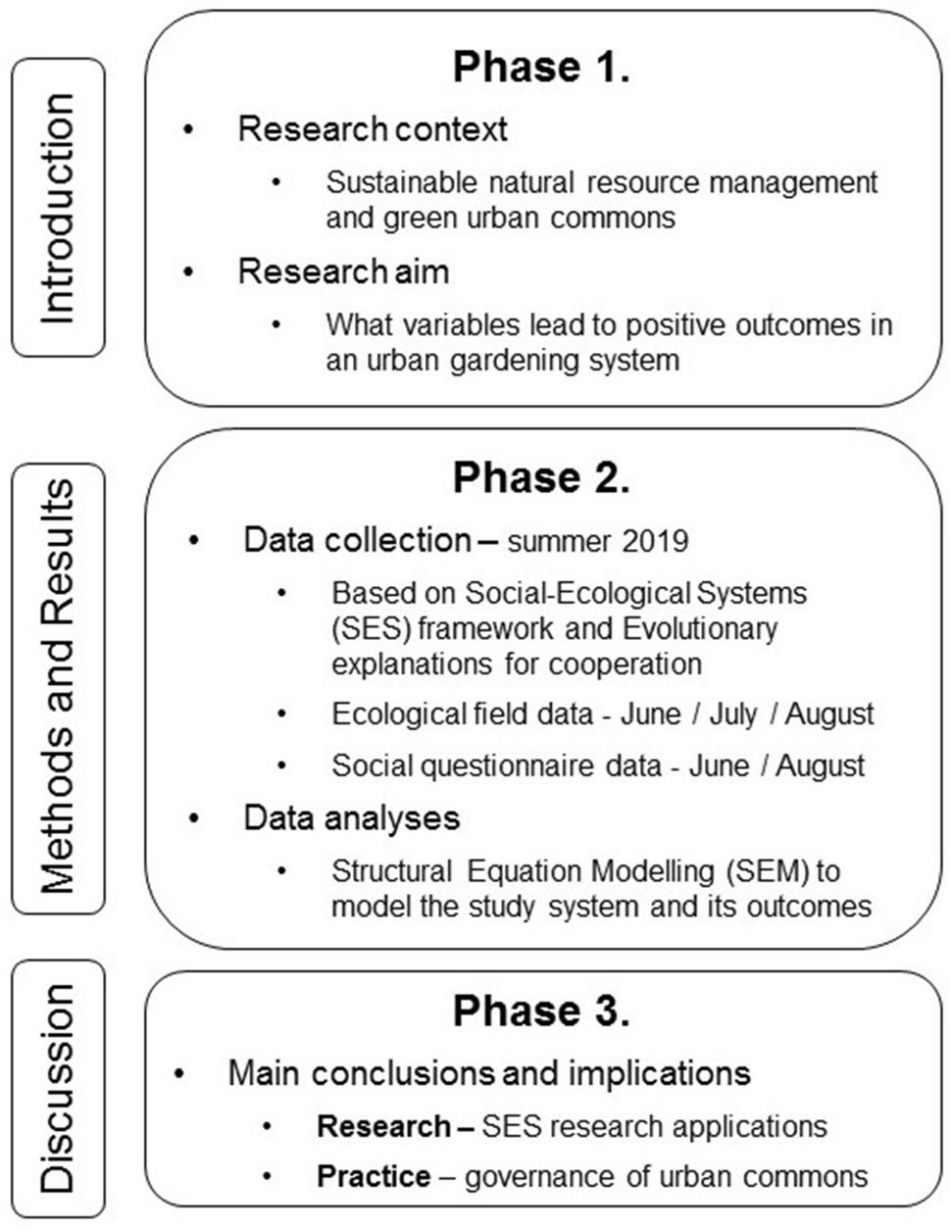
A research flowchart of the study.

To examine these questions, we adhere to Structural Equation Modeling (SEM)^45^ that allows the construction of unobserved latent variables for the scientific constructs of interest (for a flowchart of the research activities see Fig. 2). The few occasions SEM has been used successfully when studying SESs support the method`s applicability^46,47^. First, we perform a confirmatory factor analysis (CFA) to evaluate whether the proposed two latent factors, i.e. self-perceived and ecological outcomes, can be reliably measured by the specific indicator variables recorded here, and whether these two are associated. The outcomes by definition consist of several aspects^3^ and can be measured differently depending on the context^18^. Their modelling as latent variables enables us to include multiple measures of scientific constructs in one model and increase our understanding of the relative importance of these different measures. Second, we construct a full SEM to explore which variables belonging to the different SES framework and evolutionary theory subsystems are associated with the self-perceived and ecological outcomes (Fig.1).

## Results

### Garden boxes in Turku

In 2019 there were 664 garden boxes in 228 different locations in the urban area of the city of Turku. We collected data from two sources, field inventories and questionnaires, and combined them for the analyses resulting in a sample size of 121 gardener locations. All the variables used in the analyses, with a full description and expected associations, are found in Table 1.

### Measurement model for ecological and self-perceived outcomes

**Figure 3:**
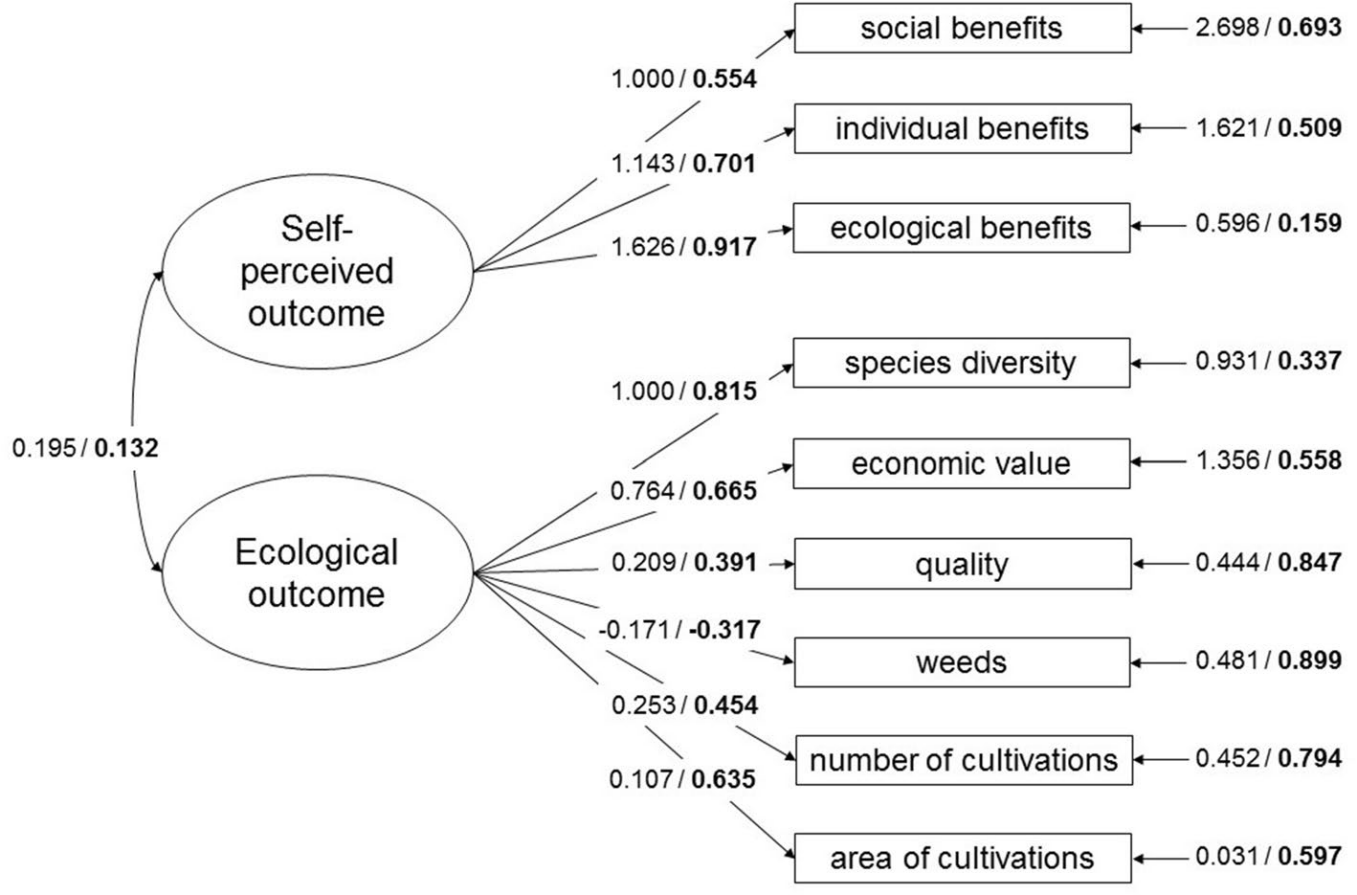
The model for ecological and self-perceived outcome –latent variables and their reflective indicators in an urban gardening system. The covariance between the latent variables, factor loadings for each reflective indicator (all significant) and error terms for the reflective indicators are presented (unstandardized estimates / standardized estimates bolded) (see Table S5 online for p-values and standard errors for each estimate). The error terms for the reflective indicators are shown after the arrows pointing to the indicators and they signify the variance not explained by the respective latent variable they are loading onto.

First, we applied CFA to construct a measurement model with ecological and self-perceived outcomes as latent variables. The latent variables were correctly measured by their indicators (Fig. 3). We investigated how well the model fits the observed data by a global fit of the model^48^. The models can be over-rejected due to our relatively small sample size, and we used Swain`s correction factor (in this study = 0.965) to correct the model fit indices. The Swain corrected global fit of the CFA model to the data was acceptable (chi-square value = 35.070, df = 26, *p* value = 0.110, RMSEA = 0.056, RMSEA 90 % C.I. = 0.000 / 0.099, CFI = 0.946). All the indicators had significant loadings onto their latent variables (*p* value < 0.005).

A more positive ecological outcome was associated with an increase in species number, economic value, quality, number and area of cultivations, and a decrease in weeds (Fig. 3). A more positive self-perceived outcome was associated with an increase in social, individual and ecological benefits (Fig. 3). The standardized factor loadings indicate that the most important indicators for the ecological outcome were species number, economic value and area of the cultivations, respectively. For the self-perceived outcome, the self-perceived ecological benefits had the highest, individual benefits the second-highest, and social benefits the lowest standardized factor loading. Contrary to our hypothesis, the self-perceived outcome and ecological outcome were not significantly correlated (correlation = 0.132, *p* value = 0.385).

### SES framework and evolutionary subsystems of importance in urban garden boxes

**Figure 4:**
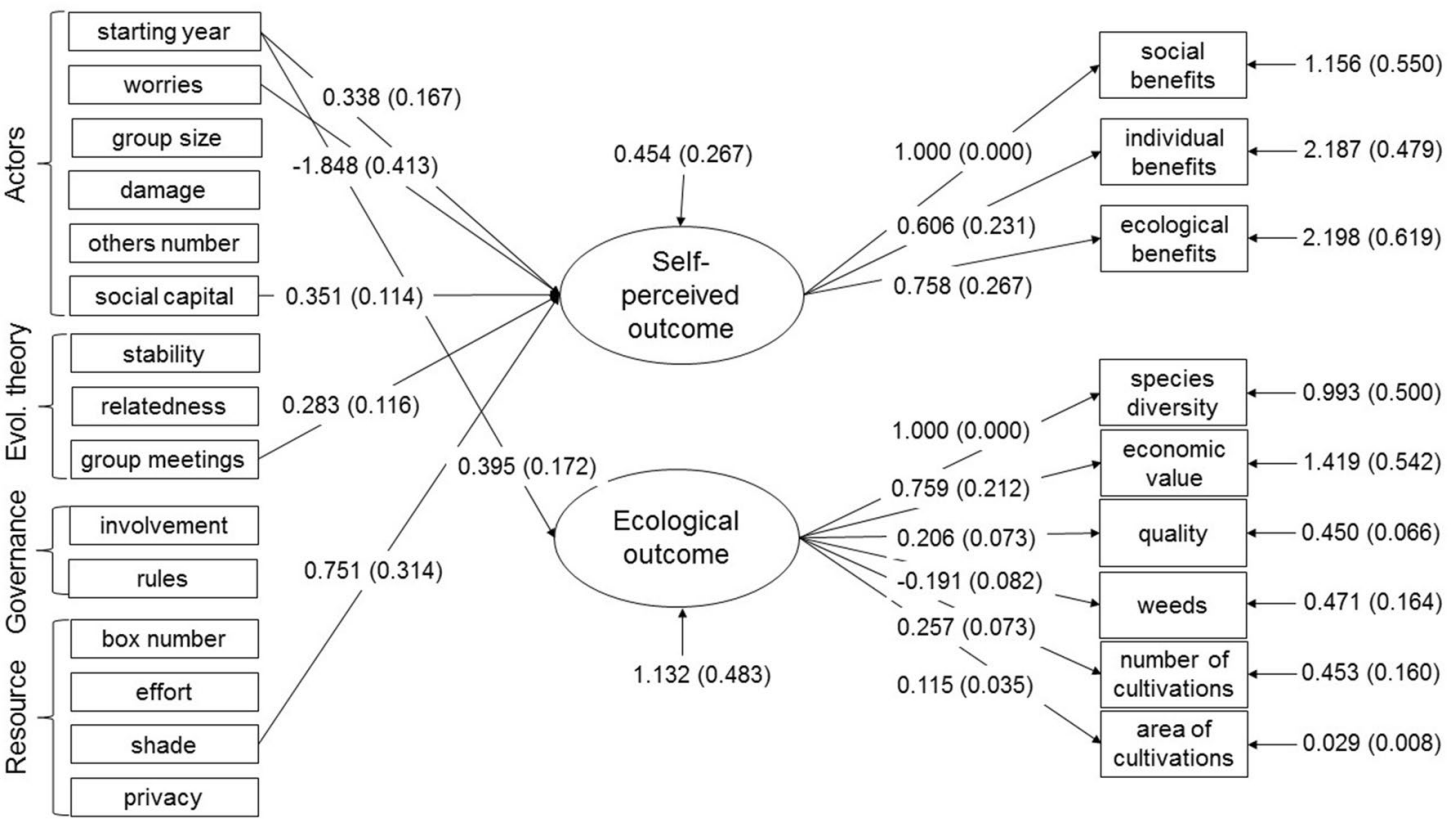
The SEM results for the variables belonging to the sub-systems Actors, Governance, Resource and Evolutionary theory, predicting the two latent variables Self-perceived and Ecological outcomes described by their reflective indicators. The regression coefficients presented by arrows pointing to the latent variables show the significant (*p* value < 0.05) associations between the predictors and the outcomes (note that the non-significant predictors were not omitted from the model, see Table 2). The estimates presented are unstandardized estimates and their standard errors are presented in brackets. Error terms signifying the variance not explained by the model are included for latent variables and the reflective indicators.

The SEM investigated if variables belonging to the actors, resource, governance, or evolutionary theory subsystems were associated with self-perceived and/or ecological outcomes (Fig. 4, Table 2). In the model, the global model fit indices are not available due to multiple imputation method to handle missing values (see methods^49^). However, the aspects indicating reliable estimates are met, namely model convergence and small standard errors^50^. Starting year, a variable belonging to the actors subsystem, associated positively with both of the outcome latent variables, meaning that their values increased when box gardening was more recently started. Social capital (actors subsystem) associated positively with self-perceived outcome. Worries (actors subsystem) was negatively associated with self-perceived outcome. If people had answered they were considerably worried if something would happen to their cultivations over summer, the less outcome was obtained. Group meetings, a variable belonging to the evolutionary theory subsystem, associated positively with self-perceived outcomes. When the group interacted more frequently, the outcome was higher. Shade from the resource subsystem associated positively with the self-perceived outcome. If the group`s boxes were located in a sunnier spot, the outcome was higher. Some other predictors belonging to the concept actors were quite close to significant (Table 2). Larger groups obtained more self-perceived outcome (group size: *p* value = 0.063) and a respondent who reported damage obtained more self-perceived (damage: p-value 0.085) as well as ecological (damage: *p* value = 0.090) outcome.

The variance explained for the ecological outcome latent variable was modest, 36.3% (S.E. = 11.2%, *p* value = 0.001), but the SE model explained the variance in the self-perceived outcome latent variable well, 84.8% (S.E. = 10.7%, *p *value < 0.001).

**Table 2:** SEM Results for the Structural Model (Fig. 3).

Latent variable	Independent variable	Estimate	S.E	*p* value
Self-perceived Outcomes ON	**Actors**			
	Starting year	**0.338**	0.167	**0.043**
	Worries	** − 1.848**	0.413	**0.000**
	Group size	0.235	0.126	0.063
	Damage	0.833	0.484	0.085
	Others number	0.042	0.062	0.502
	Social capital	**0.351**	0.114	**0.002**
	**Evolutionary theory**			
	Stability	− 0.543	0.376	0.149
	Relatedness	0.699	0.451	0.121
	Group meetings	**0.283**	0.116	**0.014**
	**Governance**			
	Involvement	0.364	0.544	0.504
	Rules	0.093	0.425	0.827
	**Resource**			
	Box number	0.040	0.105	0.705
	Effort	0.017	0.353	0.961
	Shade	**0.751**	0.314	**0.017**
	Privacy	0.206	0.347	0.551
Ecological Outcomes ON	**Actors**			
	Starting year	**0.395**	0.172	**0.022**
	Worries	0.155	0.361	0.667
	Group size	− 0.061	0.054	0.260
	Damage	0.951	0.561	0.090
	Others number	0.040	0.073	0.587
	Social capital	0.182	0.134	0.174
	**Evolutionary theory**			
	Stability	0.478	0.422	0.256
	Relatedness	− 0.074	0.311	0.812
	Group meetings	0.071	0.137	0.607
	**Governance**			
	Involvement	0.086	0.680	0.899
	Rules	− 0.222	0.491	0.651
	**Resource**			
	Box number	− 0.042	0.086	0.626
	Effort	− 0.199	0.346	0.566
	Shade	− 0.326	0.299	0.277
	Privacy	− 0.071	0.304	0.814

This table presents the unstandardized estimates for the regression coefficients, their standard error and *p* value for all the independent variables predicting each latent outcome variable in the urban gardening system. The significant values (p < 0.05) are in bold. They signify a meaningful predicting power of the independent variable on the latent variable when including all the other variables and the covariances between the independent variables in the model.

## Discussion

We apply the SES framework and evolutionary theory in a novel way to gain insight into the governance of urban garden boxes by self-organized gardening groups in the city of Turku in Finland. We find that (1) self-perceived and ecological outcomes in this urban gardening system are not associated. Against expectations^26,27,33^, gardeners who succeed in terms of ecological outcomes, i.e. good quality produce and species diversity do not necessarily self-evaluate to have succeeded ecologically, socially, and individually. We also find that (2) particular characteristics of the gardening group (starting year, worries and social capital) are associated with changes in the outcomes (especially self-perceived) in urban gardening. In contrast, few of the aspects related to governance system and resource associate with the outcomes. In addition, (3) we find that relatedness between resource users, a potential evolutionary explanation for cooperation, was not relevant in our case. However, repetition of interactions indeed positively influences the self-perceived outcomes of urban gardening, suggesting that reciprocity is more important than kin selection in this context.

We first examined whether the multiple observed variables included in the study adequately measured self-perceived and ecological outcomes in the urban garden box resource system. The SES case studies rarely include more than one measure at a time, even though research would benefit from including multiple references^36,43,51^. Several measures describe more genuinely the multidimensional and complex reality of natural resource systems. Our model fitted the data satisfactorily and all our measured variables had significant loadings onto their latent variable. In urban gardening research the typical ecological performance measure is either the species diversity or the gardeners` evaluation of their harvest, which simplifies the numerous aspects related to the harvest or yield^32,37,43^. Socio-economic performance has been measured for example, by self-organization, livelihood, gender equity or different socioeconomic factors^18,34,36^. As the outcome measures are so various we encourage future studies of urban commons to include multiple measures of outcomes or benefits to arrive at a more holistic understanding of the system^51^.

The SEM approach offers sound statistical benefits when using many indicator variables to describe a latent, because it accounts for the measurement error, thereby providing more realistic estimates and acknowledging the difficulty in measuring the outcomes exhaustively^45^. Therefore, the conclusions based on latent variables are more general. When considering SES research, different studies can measure and compare the same concepts but operationalize them differently depending on the data and study aims. Lastly, SEM approach allows investigating the relative importance of different measures for the outcomes. Considering our latent variables, ecological benefits (to create biodiversity, self-sufficiency, to beautify the area, fresh vegetables) was the most important measure for the self-perceived outcome and species diversity was the most important measure for the ecological outcome.

Against expectations, ecological and self-perceived outcomes did not correlate significantly^27,32^. Therefore, independently of the gardening success, the gardener can report to receive e.g. much or little quality time with friends and family, mental relaxation, and fresh vegetables. It seems that the importance of the physical resource is lower than expected^43^ and the immediate human needs in this case might be fulfilled even when the ecological outcome is low. The importance of the resource for livelihood has been found to increase its sustainability^36^, which can partly explain the loss of connection here. The relationship between social and ecological aspects in social-ecological systems is complex and studies have found both positive, negative and no correlations between outcome variables^36,40,51^. As we were able to evaluate objectively the ecological outcome and compare it to the self-perceived one, our findings imply that people governing an urban common might consider they have reached satisfactory ecological benefits even though in reality this is not the case. However, our study sample is small and further work is needed to confirm if similar dynamics exist in other natural resource systems.

Interestingly, the self-evaluated ecological benefits were found to best measure the self-perceived outcome while still being uncorrelated with the ecological outcome. This contradiction suggests the need to strengthen the link between the local biodiversity outcomes and people`s perception of them when designing urban commons governance. Local species diversity is found to be connected to the ecological resilience in cities, which is one of the main goals of the gardening projects^12^. Based on our results, we can recommend that when the ecological outcomes are monitored in practice, it should not only happen through self-evaluation. Previous studies indicate that gardener motivations and knowledge influence e.g. the species diversity and therefore information considering these aspects should be available for gardeners to improve ecological outcomes^32,38^. The complex relationship between the outcomes points out the unfortunate (but not unexpected) fact that the immediate human needs often differ from sustainable ecological conditions^1^.

We demonstrate that SEM can be used to localize what are the most important variables associated with changes in outcomes in an urban garden box system. However, to maintain our sample size, we performed multiple imputations, which prevents us to study global fit indices for the model. First, against our expectations, members in gardening groups that started a longer time ago (starting year) reach less self-perceived as well as ecological outcomes. Previously it has been found that the gardening experience increases the total yield^52^ and we assumed that the knowledge gained through gardening years would have a positive influence on outcomes^3,41^ but, perhaps, the enthusiasm of starting a new “hobby” might outweigh the experience. The rest of the significant associations were all related only to the self-perceived outcome. Gardening group members who obtain more social capital (help, positive feedback and networking) over the summer feel that they receive more outcomes, similarly to earlier studies related to community gardens where longer collective action as a positive outcome correlates strongly with social cohesion and trust^43^. The finding emphasizes the social importance of the activity, which may be generalized to a wider natural resource governance^53^.Gardeners who worry about damage such as vandalism, theft or animal damages happening to their cultivations (worries) evaluate to receive less from the activity, as assumed. The variable was placed under the “past experiences” of the actors sub-system but it could be also related to the “mental models and knowledge of SES”^3^. The worries were stated at the beginning of summer, meaning that they do not necessarily associate with actual experiences but rather with gardeners` negative attitudes. The open and public location can create uncertainty related to risks and gardeners` control, which influences the outcomes^44^.

We found that the sunnier place the box was located in, the more self-perceived outcome was received. It was the only significant predictor from a SES framework sub-system resource, and, associated with the productivity of the system (RS5)^3^. Previously it has been found that the size of the resource system i.e. garden size or box number in our case strongly influences plant species richness, which was not found in our study^38^. Taken together, our analyses suggest that SES framework variables mainly related to the level of the individuals cooperating in governing the resource (actors) are of importance in our urban gardening system, especially in terms of self-perceived outcomes.

We were unable to find many associations between the ecological outcome and our predictor variables, which indicates low importance of the physical harvest as a motivator, supporting previous findings^43^. We also found that variables from governance subsystem, involvement indicating the fair division of labor, and rules were not significant predictors, contrary to many other natural resource systems^15^. However, in community gardens monitoring and sanctions are not assumed to be important as free-riding in the form of the physical resource is not perceived so negatively because of the low importance of the harvest^43^. Less than 40 % of variance in ecological outcome was explained in our models while for the self-perceived outcome the number was over 80 %. Therefore, we encourage future studies to include additional predictors to reach better understanding of the aspects mediating ecological outcomes^13,32,38^. The literature on urban gardening is largely based on community gardens and domestic gardens, and these kinds of urban gardens are markedly different from garden boxes. One clear difference is that community and domestic gardens are organized and governed in a different way, and when different motivations drive the participants, the dynamics also change^14^.

In previous studies factors such as gender, region of origin, neighborhood, motivations and time spent gardening have been found to influence changes in plant species diversity^32,38^. Our findings are partly based on questionnaire data, where the non-respondents can differ from the observed respondents. For example, non-respondents can be gardeners not experiencing positive outcomes, and their exclusion could weaken the links between the predictors and outcomes. The garden box scheme, run by the City of Turku, collects no personal information and we therefore lack background knowledge on the profile of urban gardeners in this scheme. Our survey was distributed in multiple ways contacting all the gardeners (directly to registered users, social media and by leaflets placed at the garden boxes); we achieved a high response rate of about 50%^54,55^. Arguably, this high response rate safeguards the findings from strong biases due to non-responsiveness. However, the possible bias toward overrepresentation of the successful respondents prevent us from making clear conclusions about why and when gardeners fail entirely in this activity. Clearly, however, subsequent studies of gardening box systems would benefit from including more socio-demographic characteristics^38^.

Our third study question considered if the variables relatedness, stability of groups or repetition of interactions, derived from evolutionary theory would be relevant in our study system^22,23^. None of them were found to associate with the ecological outcome, but we were able to show that more frequent interactions significantly influence positively the self-perceived outcome. Relatedness, an important aspect explaining cooperative behavior in non-human world, is thus not notably significant when controlled by stability and group meetings^4^. However, in line with other studies showing how indirect fitness benefits drive cooperation in humans^20,21^ more self-perceived social, individual-level and ecological benefits were received in groups where members interact more frequently with each other. Furthermore, as emotions and other psychological mechanisms seem to mediate human cooperation in many contexts, it is possible that self-perceived benefits, even if dissociated from biological fitness, could foster sustainable resource use in many SESs^56^. The presence of face-to-face communication is known to be an important variable in forming sustainable resource governance^5^ and repeated interactions increase collective action in community gardens^2,43^. When group members meet more often, it can improve mutual trust, division of labor and efficiency facilitating collective action^24,25^. There`s also more opportunity for social control, which eventually leads to the correlation of strategies within groups and makes the actors more cooperative^4^. Due to the high importance of face-to-face communication in literature and our finding being in line with other studies, we can suggest that frequent interactions is a major indicator for working natural resource governance^25,57^.

## Conclusions

Our findings suggest that the decoupling of ecological and self-perceived outcomes stems from the aspect that they mainly are impacted by different variables. Due to limited scope of our study, we note that further work in similar systems is needed to establish the finding and the causal linkages. However, a combination of self-evaluated as well as objectively measured outcomes seems relevant in analyzes of natural resource governance. In particular in urban gardening projects, where both social and ecological resilience of cities is a key aim, the disconnection highlights the need for more ecological awareness^13,37^.Although urban garden boxes have only a small effect on urban resilience, their cumulative local influence can be higher through well-working spatial and temporal coordination^13^. Our results both have practical value for urban commons governance, and contribute theoretically to SES research by highlighting perspectives from evolutionary explanations of cooperation^4^. Even if slightly limited by the small sample size, we were able to model the self-perceived and ecological outcomes of the activity as latent variables including multiple measures, which has a great advantage when comparing different systems and case studies^19,35,46^. Our results emphasize the social importance of the activity, and highlight the importance of frequent interactions to positive outcomes in natural resource governance^24,25,57^. Diminishing outcomes with time suggest it’s important to carry out long term studies in order to understand the motivations of actors and their effects on the ecological and social impacts. Despite their differential effects, the variables we here identify correlating with outcomes are mostly aspects pertaining to the actors in the system. We, therefore, believe our findings exemplify the importance of understanding natural resource governance systems at a very low “grassroot” level.

## Methods

### Urban garden boxes in the public space

**Figure 5:**
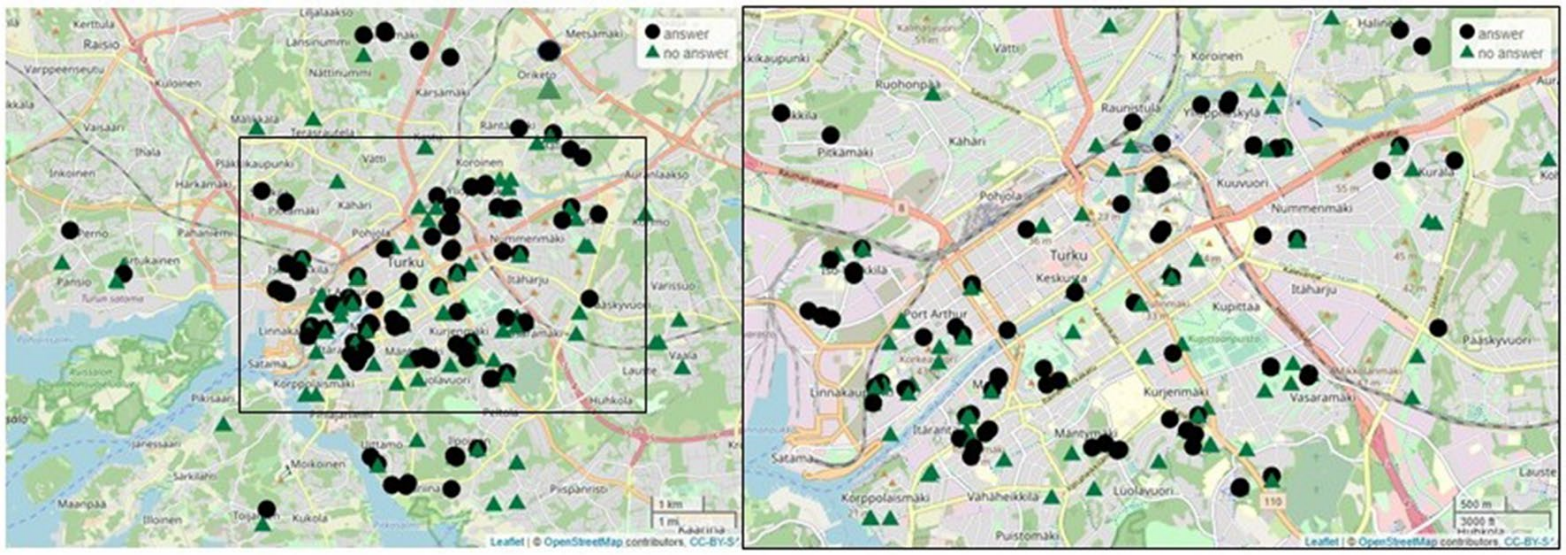
Map of Turku city area presenting the locations of the urban box gardening groups. The boxes were distributed around Turku quite evenly (on the left all the locations), however concentrating on the city center (augmented map on the right). The black circles represent groups, who answered the questionnaire and the green triangles the groups, who didn`t answer the questionnaire.

This study was conducted in 2019 in the urban area of a medium-sized city of Turku (population about 200 000) in Southwestern Finland and focused on a group of urban box gardeners enrolled in the city`s gardening program. The city provides one or more garden boxes (each 1 m^2^ in size) and soil for free for people who volunteer to take care of them. The gardeners self-organize, can cultivate whatever they desire and the boxes are placed where the gardeners choose as long as they are on public land. Each year the demand exceeds the supply as the city distributes only a limited number of boxes. In 2019 there were 664 garden boxes in 228 different locations (Fig. 5). The boxes were located widely around Turku area, however mainly in the city center.

### Modifying the SES framework for urban garden boxes

First, we considered which variables in the second-tier variable list of the SES framework^3,15^ are potentially relevant to urban gardening outcomes (Table 1; a complete list see Supplementary Table S1). The variables of the framework are not all intended to be used in every case study^18^: the addition or exclusion of the variables as well as their explicit operationalization depends on the context. We were especially interested in actors directly involved in resource governance, in their decision making and local conditions for resource sustainability. To this end, nine SES variables and three evolutionary variables were chosen to predict nine outcomes. We based our data collection on this selection. Some of the SES variables were operationalized by one measured variable (e.g. size of the resource system = box number), while some were operationalized by a higher number of measured variables (e.g. number of relevant actors = group size and others number).

### Data collection

Data was collected by field inventories and two questionnaires sent to the gardeners in summer 2019. The field inventories aimed to collect variables objectively measuring the ecological outcome and the SES resource sub-system. The field data was collected from all of the urban gardening boxes (664) in all locations (228) in the Turku area. The garden boxes were inspected three times five weeks apart (June, July and September). The average values per box over the summer are used in the analyses. During each visit, to assess the ecological outcome, the cultivated species were identified and counted, their quality and area cover were evaluated and the area covered by weeds was estimated (Table 1). In addition, the garden box location was evaluated for the average shade, privacy, and possible damage to the cultivations or boxes (Resource system, Table 1). Extra effort (RS4 human-constructed facilities) was recorded for the group if any kind of additional effort was observed, as this was seen as a sign of motivation^32^.

To elaborate the economic benefits of gardening, the estimated economic value of cultivations per box was included as one measure of the ecological outcome. While this is atypical in SES research, it is an additional aspect of gardening success. After each field inventory, an approximate sales price (€) for buying the gardened product in a shop in Turku (foodie.fi, 2019) was computed. The price €/piece was calculated for each plant and the price €/kg was calculated for the species according to their area (m^2^) and information acquired of the average yield kg/m^2^ for each species^58^. In addition, the final economic value was multiplied by the quality of each species.

Two questionnaires were sent to the gardeners, one before and one after summer 2019, using an electronic questionnaire (made in Webropol 2.0 online surveys) (see Supplementary Table S2 & S3 online). All the enrollees were contacted through the city program`s gardener registry. Overall, 121 gardeners answered at least one survey, which was 53.1% of all the registered groups. The response rate is considered acceptable for representation (60 +/− 20^54^) and the analyses were performed to these units^55^. As the boxes are applied by an electronic form, we can assume that an electronic questionnaire is accessible and that the gardeners have an equal chance of answering. We enhanced the response rate by advertising the survey in the program`s Facebook group as well as distributing a short information package at each box. There is no information considering the demographic attributes of all the enrollees, nor can we assume the gardeners to represent the attributes of the population of Finland or Turku in general. Therefore, a limitation for our study is the inability to investigate how the respondents differ from non-respondents in these aspects. As all the gardeners were approached and the response rate is high, we can assume that our data covers the gardener group sufficiently. In addition, there is no visible geographic bias in the representation of the respondents compared to the non-respondents and the questionnaire answers cover the areas where boxes are found (Fig. 5). In addition, the respondents represent adequately the starting years of all the groups (the proportional numbers: overall / survey in 2019: 39,47% / 35,83%, 2018: 21,49% / 25,83%, 2017: 15,79% / 10,83% and 2016: 23,25% / 27,50%).

The questionnaires collected data to measure the two variables belonging to the subsystem governance (rules, involvement), six variables belonging to the subsystem actors (group size, others number, starting year, damage, worries, social capital), three variables belonging to the subsystem evolutionary theory (group meetings, relatedness, stability) and three variables measuring self-perceived outcome (social, individual and ecological benefits) (see all questions Tables S2 & S3 online, all variable definitions Table 1). Next, we describe some variables that may need further clarification. The relatedness was estimated as the respondent`s description of their family connections in the group. They stated if people in their gardening group were their family members (children and/or parents), other family members, friends, neighbors, or others. The social capital was formed as a composite consisting of questions about reciprocity, feedback and social networks related to the activity^25,53^. The after-summer questionnaire asked about 14 different benefits potentially obtained from the garden box activity to measure the self-perceived outcome. This list of benefits was partly based on the self-identified expectations from the survey before the summer, and partly on previous research^26,31,33^. The respondents were asked if they received these benefits during the summer and hence constituted a self-evaluation of the outcomes. 12 of the benefits were grouped into 3 categories depending on if they were related to individual, social or ecological benefits. To have 4 in each category, overall happiness (not possible to group under one category) and new acquaintances (not an expected benefit) were excluded from the analysis. The benefits were not studied separately due to the limitation of our small sample size.

The information collected through the field inventories was connected to the information collected from the surveys by a name the group had stated for themselves when registering to the city. The surveys were planned for gardening groups, but some respondents reported taking care of the boxes alone. There were questions (rules, involvement, group meetings, relatedness and stability), which could not be answered sensibly by a one-person group, and therefore these answers had to be excluded from these questions (NA).

### Modeling the urban gardening resource system using structural equation modeling

Structural equation modeling (SEM) combines factor analysis and path analysis into a single statistical framework^31^. The main advantage of SEM is the possibility to construct multiple-indicator latent (unobserved) constructs that represent constructs of scientific interest while accounting for measurement error. Our analyses were primarily based on an a priori hypothesized but simplified social-ecological framework (Fig. 1). First, we applied confirmatory factor analysis (CFA) to construct a measurement model with ecological and self-perceived outcomes as latent variables, each measured by reflective indicators (i.e., indicators are assumed to be causally affected by the underlying latent variable)^45^. The ecological outcome in the urban garden boxes was measured by six indicators recorded during the field inventories; the number of species per box (the marker variable used to scale the latent variable by fixing its loading to 1 and intercept to zero), number of cultivations per box, area of cultivations per box, average quality, average economic value and coverage of weeds per box (Table 1). The number of cultivations was divided by 100 and coverage of weeds and economic value were divided by 10 to avoid too large unique variances of the indicators. The self-perceived outcome of garden box activity was measured by three indicators quantified in the questionnaire; i.e. social benefits (the marker variable), individual benefits and ecological benefits (Table 1). All these indicators were continuous variables (Table 3). The indicators measuring each latent variable correlated with each other (Tables 4 and 5), as required for reflective indicators assumed to be causally linked with the same latent^59^. To compare the indicators` relative differences the fully standardized parameter estimates for the factor loadings were reported.

**Table 3:** Descriptive statistics for the variables used in the analyses.

Variable name	Mean	Median	Min	Max	S.D	% of missing	Variable type
**Self-perceived outcome**							
Social benefits	2.72	3	0	8	1.97	41.32	Continuous
Individual benefits	4.83	5	1	8	1.78	41.32	Continuous
Ecological benefits	4.38	4	0	8	1.94	41.32	Continuous
**Ecological outcome**							
Species diversity	3.22	3	0.60	8.33	1.66	8.27	Continuous
Economic value	1.47	1.01	0.001	11.77	1.56	8.27	Continuous
Quality	3.87	4	2	5	0.73	8.27	Continuous
Weeds	0.43	0.2	0	4.25	0.73	8.27	Continuous
Number of cultivations	0.73	0.46	0.03	5.21	0.76	8.27	Continuous
Area of cultivations	0.44	0.43	0.06	1.30	0.23	8.27	Continuous
**Resource**							
Box number	2.71	2	1	15	2.44	0.83	Continuous
Effort	0.38	0	0	1	0.49	8.27	Binary
Shade	0.65	1	0	1	0.48	8.27	Binary
Privacy	0.45	0	0	1	0.50	8.27	Binary
**Governance**							
Rules	0.48	0	0	1	0.50	63.64	Binary
Involvement	0.75	1	0	1	0.44	64.46	Binary
**Actors**							
Group size	3.04	2	1	23	3.22	0	Continuous
Others number	2.45	2	0	15	2.92	41.32	Continuous
Starting year	1.7	2	0	3	1.22	0.83	Continuous
Damage	0.23	0	0	1	0.42	60.33	Binary
Worries	0.46	0	0	1	0.50	17.36	Binary
Social capital	3.41	3	0	8	1.71	41.32	Continuous
**Evolutionary theory**							
Group meetings	4.31	5	1	6	1.61	60.33	Ordinal
Relatedness	0.40	0	0	1	0.49	0.83	Binary
Stability	0.28	0	0	1	0.45	41.32	Binary

This table reports descriptive statistics on the outcome and predictor variables. The data for the 121 gardening groups consists of information from different sources (field, two questionnaires), which leads to varying amount of missing values reported here. The variables were treated in the analyses as continuous or binary. See the description for each variable in Table 2.

**Table 4:** Correlation matrix for self-perceived outcome indicators.

Variable name	Social benefits	Individual benefits	Ecological benefits
Social benefits	1.000		
Individual benefits	0.388	1.000	
Ecological benefits	0.508	0.643	1.000

Correlation matrix for the continuous reflective indicators (social, individual and ecological benefits) measuring the latent variable Self-perceived outcome. All correlations are significant (*p* value < 0.05).

**Table 5:** Correlation matrix for ecological outcome indicators.

Variable name	Species diversity	Economic value	Quality	Weeds	Number of cultivations	Area of cultivations
Species diversity	1.000					
Economic value	0.541	1.000				
Quality	0.318	0.260	1.000			
Weeds	− 0.258	− 0.211	− 0.124	1.000		
Number of cultivations	0.370	0.302	0.177	− 0.144	1.000	
Area of cultivations	0.517	0.248	0.248	− 0.201	0.288	1.000

Correlation matrix for the continuous reflective indicators (species diversity, economic value, quality, weeds, number of cultivations and area of cultivations) measuring the latent variable Ecological outcome. All correlations are significant (*p* value < 0.05).

The two latent variables were modelled simultaneously to investigate their association. The global fit of this model to the data was evaluated by a chi-square test and the following fit indices: root mean squared error of approximation (RMSEA) and comparative fit index (CFI)^48^. In the chi-square test, the *p* value over 0.05 signifies acceptable model fit, since the null hypothesis is testing whether the model-implied variance-covariance matrix equals that of the observed data. The cut-off values of fit indices for an acceptable model fit are for RMSEA =< 0.06 and CFI > 0.90^48^. The models for small sample sizes can be over-rejected and therefore we used the Swain`s correction factor (in this study = 0.965) to evaluate model fit using the R function swain.

Second, in the same framework, we regressed the variables from the actors, governance, resource, and evolutionary theory subsystems on the two latent variables, self-perceived and ecological outcomes to investigate which of the individual variables would statistically predict the outcomes. Most of the predictor variables were coded as binary for the analysis, except for two ordinal and four continuous variables, which in the analyses are both treated as continuous (Table 3). The variables regressed to the latent variables did not present significant correlations with each other, showing that multicollinearity was not an issue here (Table S4 online)^59^. The global model fit indices are not available for the second model due to multiple imputations to handle missing values (see below^49^).

Our data contained non-normal continuous response variables (i.e. indicators), and therefore we used a robust maximum-likelihood estimator (MLR)^49^. Our sample size (n = 121) is relatively small for SEM analysis, which can cause convergence issues and bias in parameter estimates^50^. The rule of thumb for a sufficient sample size of 10 observations per 1 free parameter was not realized for the measurement model (114:28) or the second model (114:58)^45,60^.However, it has been found that a small sample size might not face these issues when factors are defined by 3 or more indicators, 5 or fewer latent variables are created and an ML estimation is utilized^61,62^. In addition, when the model converges within a small number of iterations (max 50) and parameter estimates are reasonable, including relatively small standard errors, the results for local parameter estimates are not biased owing to a small sample size^50^. In this study, all of the above-mentioned requirements were fulfilled.

The CFA model excluded 7 observations as they contained missing data in all of the variables. Missing data for independent variables in the second model was dealt by multiple imputation (MI)^49^. In MI, multiply imputed datasets are created where the missing values are substituted, analyses are performed for each dataset and the results are combined. The main advantage of MI is to account for the uncertainty of estimating missing data by considering the variability between the datasets. To assure the reliability of the results (point estimates and their SEs) in the presence of over 60% missing data in some variables and maintain statistical power for our sample size, 100 imputed datasets were generated for our analyses^63^. MI can be used to obtain reliable results for non-normal data, small sample size as well as high proportions of missing data^64^. The missing values for indicators (treated as dependent variables in all models) were not imputed in the datasets, but the model was estimated for each imputed dataset using full information maximum likelihood (FIML)^65^. Analyses were conducted using Mplus (Version 8.5)^49^. Mplus code for performing the MI and models are provided in the supplementary material (Fig. S1a–c. online).

### Ethical approval

This study was conducted by the University of Turku. It collected opinions from human participants in a garden box scheme operated by the City of Turku by means of a questionnaire amongst garden box participants. Participation in the study was voluntary. A link to the survey was sent out to participants directly by the City of Turku without involvement of the University of Turku. In addition, links to the survey was distributed via social media and was advertised as a flyer left anonymously on the garden boxes. The questionnaire did not contain questions allowing identification of individuals. As a consequence, the information for this study was gathered in a manner where no personal information was collected by the University of Turku that would allow identification of individual human participants. The study does not fall under the Declaration of Helsinki. The University of Turku adheres to the Finnish national ethical standards of research with human participants, as specified in the Code of Research Conduct of the Finnish National Board on Research Integrity (TENK) (Decree 1347 of 15 November 1991). The study protocol and data management are all in accordance with the EU General Data Protection Regulation (GDPR) principles. All participants were provided with the required information about the project, data protection and participant rights through a GDBR-compliant privacy notice. Informed consent was obtained from all participants when they submitted the information and agreed to participate in the project. The responding to the questionnaire is considered to be written informed consent as the respondents were fully informed by the privacy notice and the questionnaire did not collect any personal information nor include sensitive questions. The data provided can be used for publication. Given our study followed the above outlined criteria, no ethical review is needed according to the Finnish ethical guidelines when review is required in human research (https://tenk.fi/sites/default/files/2021-01/Ethical_review_in_human_sciences_2020.pdf). The guidelines for ethical review in research with human participants are intended for research designs where ethical review is not regulated separately in the Medical Research Act (488/1999).

## Supplementary Information

Supplementary Information.

## Data Availability

The datasets generated during and/or analyzed during the current study are available from the corresponding author on reasonable request.

## References

[1] William CC, Levin SA (2009). Toward a science of sustainability. Toward Sci. Sustain..

[2] Ostrom E (2007). A diagnostic approach for going beyond panaceas. Proc. Natl. Acad. Sci. U.S.A..

[3] Ostrom E (2009). A general framework for analyzing sustainability of social-ecological systems. Science.

[4] Rankin DJ, Bargum K, Kokko H (2007). The tragedy of the commons in evolutionary biology. Trends Ecol. Evol..

[5] Ostrom E (2009). Understanding institutional diversity.

[6] Bodin Ö (2017). Collaborative environmental governance: achieving collective action in social-ecological systems. Science.

[7] Nagendra H, Ostrom E (2014). Applying the social-ecological system framework to the diagnosis of urban lake commons in Bangalore, India. Ecol. Soc..

[8] Leslie HM (2015). Operationalizing the social-ecological systems framework to assess sustainability. Proc. Natl. Acad. Sci. U.S.A..

[9] Cumming GS (2020). Advancing understanding of natural resource governance: a post-Ostrom research agenda. Curr. Opin. Environ. Sustain..

[10] Perrotti D, Hyde K, OteroPeña D (2020). Can water systems foster commoning practices? analysing leverages for self-organization in urban water commons as social–ecological systems. Sustain. Sci..

[11] Radywyl N, Bigg C (2013). Reclaiming the commons for urban transformation. J. Clean. Prod..

[12] Colding J, Barthel S (2013). The potential of 'Urban Green Commons' in the resilience building of cities. Ecol. Econ..

[13] Aronson MFJ (2017). Biodiversity in the city: key challenges for urban green space management. Front. Ecol. Environ..

[14] Fox-Kämper R (2018). Urban community gardens: an evaluation of governance approaches and related enablers and barriers at different development stages. Landsc. Urban Plan..

[15] McGinnis MD, Ostrom E (2014). Social-ecological system framework: initial changes and continuing challenges. Ecol. Soc..

[16] Taylor JR, Lovell ST (2014). Urban home food gardens in the Global North: Research traditions and future directions. Agric. Hum. Values.

[17] Binder CR, Hinkel J, Bots PWG, Pahl-Wostl C (2013). Comparison of frameworks for analyzing social-ecological systems. Ecol. Soc..

[18] Partelow S (2018). A review of the social-ecological systems framework: applications, methods, modifications, and challenges. Ecol. Soc..

[19] Herrero-Jáuregui C (2018). What do we talk about when we talk about social-ecological systems? A literature review. Sustainability (Switzerland).

[20] West SA, Griffin AS, Gardner A (2007). Evolutionary explanations for cooperation. Curr. Biol..

[21] Levin SA (2014). Public goods in relation to competition, cooperation, and spite. Proc. Natl. Acad. Sci. U.S.A..

[22] Queller DC (2011). Expanded social fitness and Hamilton's rule for kin, kith, and kind. Proc. Natl. Acad. Sci. U.S.A..

[23] Axelrod R, Hamilton WD (1981). The evolution of cooperation. Science.

[24] Ostrom, E., Ahn, T. K. & Kingdom, U. A Social Science Perspective on Social Capital. *Sociol. J. Br. Sociol. Assoc.*, 812–855 (2001).

[25] Sobel J (2002). Can we trust social capital?. J. Econ. Lit..

[26] Tornaghi C (2014). Critical geography of urban agriculture. Prog. Hum. Geogr..

[27] Chalmin-Pui LS, Griffiths A, Roe J, Heaton T, Cameron R (2021). Why garden?—attitudes and the perceived health benefits of home gardening. Cities.

[28] Feinberg A, Ghorbani A, Herder P (2021). Diversity and challenges of the urban commons: a comprehensive review. Int. J. Commons.

[29] Winkler B, Maier A, Lewandowski I (2019). Urban gardening in Germany: Cultivating a sustainable lifestyle for the societal transition to a bioeconomy. Sustain. (Switzerland).

[30] Guitart D, Pickering C, Byrne J (2012). Past results and future directions in urban community gardens research. Urban For. Urban Green.

[31] Andersson E (2014). Reconnecting cities to the biosphere: Stewardship of green infrastructure and urban ecosystem services. Ambio.

[32] Philpott SM (2020). Gardener demographics, experience, and motivations drive differences in plant species richness and composition in urban gardens. Ecol. Soc..

[33] Dunnett N, Qasim M (2000). Perceived benefits to human well-being of urban gardens. HortTechnology.

[34] Basurto X, Gelcich S, Ostrom E (2013). The social–ecological system framework as a knowledge classificatory system for benthic small-scale fisheries. Glob. Environ. Change.

[35] Villamayor-Tomas S (2020). Using case study data to understand SES interactions: a model-centered meta-analysis of SES framework applications. Curr. Opin. Environ. Sustain..

[36] Persha L, Agrawal A, Chhatre A (2011). Social and ecological synergy: local rulemaking, forest livelihoods, and biodiversity conservation. Science.

[37] Egerer MH, Lin BB, Threlfall CG, Kendal D (2019). Temperature variability influences urban garden plant richness and gardener water use behavior, but not planting decisions. Sci. Total Environ..

[38] van Heezik Y, Freeman C, Porter S, Dickinson KJM (2013). Garden size, householder knowledge, and socio-economic status influence plant and bird diversity at the scale of individual gardens. Ecosystems.

[39] Tantarimäki, S. Urbaani maatalous maankäytön ja yhteiskunnallisen tilanteen muutoksessa. *Tapaustutkimuksena Turun ja Seinäjoen taajamat. Turun yliopisto. Turku* (2003).

[40] Chhatre A, Agrawal A (2009). Trade-offs and synergies between carbon storage and livelihood benefits from forest commons. Proc. Natl. Acad. Sci..

[41] Agrawal A, Chhatre A (2006). Explaining success on the commons: Community forest governance in the Indian Himalaya. World Dev..

[42] Cinner JE (2016). Bright spots among the world’s coral reefs. Nature.

[43] Feinberg A, Rogge N, Hooijschuur E, Ghorbani A, Herder P (2021). Sustaining collective action in urban community gardens. Jasss.

[44] Cinner JE (2012). Comanagement of coral reef social-ecological systems. Proc. Natl. Acad. Sci. U.S.A..

[45] Kline, R. B. *Principles and practice of structural equation modeling*. (Guilford publications, 2015).

[46] Asah ST (2008). Empirical social-ecological system analysis: From theoretical framework to latent variable structural equation model. Environ. Manag..

[47] Allen MC, Lockwood JL, Burger J (2021). Finding clarity in ecological outcomes using empirical integrated social–ecological systems: a case study of agriculture-dependent grassland birds. J. Appl. Ecol..

[48] Hu LT, Bentler PM (1999). Cutoff criteria for fit indexes in covariance structure analysis: Conventional criteria versus new alternatives. Struct. Equ. Model. Multidiscip. J..

[49] Muthén, L. K. & Muthén, B. O. Mplus user’s guide (Version 7). *Los Angeles, CA: Author* (1998).

[50] Gignac GE (2006). Self-reported emotional intelligence and life satisfaction: Testing incremental predictive validity hypotheses via structural equation modeling (SEM) in a small sample. Personal. Individ. Differ..

[51] Agrawal A, Chhatre A (2011). Against mono-consequentialism: Multiple outcomes and their drivers in social-ecological systems. Glob. Environ. Change.

[52] CoDyre M, Fraser EDG, Landman K (2015). How does your garden grow? an empirical evaluation of the costs and potential of urban gardening. Urban For. Urban Green..

[53] Pretty J (2003). Social capital and the collective management of resources. Science.

[54] Baruch Y (1999). Response rate in academic studies-a comparative analysis. Hum. Relat..

[55] Fan W, Yan Z (2010). Factors affecting response rates of the web survey: a systematic review. Comput. Hum. Behav..

[56] Burton-Chellew MN, Ross-Gillespie A, West SA (2010). Cooperation in humans: competition between groups and proximate emotions. Evol. Hum. Behav..

[57] Andersson KP (2004). Who talks with whom? the role of repeated interactions in decentralized forest governance. World Dev..

[58] (OSF), O. S. o. F. (Helsinki: Natural Resources Institute Finland, www.stat.fi/til/satot/index_en.html, 2019).

[59] Grewal R, Cote JA, Baumgartner H (2004). Multicollinearity and measurement error in structural equation models: implications for theory testing. Mark. Sci..

[60] Kyriazos TA (2018). Applied psychometrics: sample size and sample power considerations in factor analysis (EFA, CFA) and SEM in general. Psychology.

[61] Gerbing DW, Anderson JC (1985). the effects of sampling error and model characteristics on parameter estimation for maximum likelihood confirmatory factor analysis. Multivar. Behav. Res..

[62] Teh PL, Sun H (2012). Knowledge sharing, job attitudes and organisational citizenship behaviour. Ind Manag. Data Syst..

[63] von Hippel PT (2020). How many imputations do you need? a two-stage calculation using a quadratic rule. Sociol. Methods Res..

[64] Wayman, J. C. in *Annual Meeting of the American Educational Research Association, Chicago, IL.* 16.

[65] Olinsky A, Chen S, Harlow L (2003). The comparative efficacy of imputation methods for missing data in structural equation modeling. Eur. J. Oper. Res..

[66] Tang SY (1991). Institutional arrangements and the management of common-pool resources. Public Adm. Rev..

