# Supplementary material for: Structural equation modeling reveals decoupling of ecological and self-perceived outcomes in a garden box social-ecological system
Source: Sci Rep. 2022 Apr 19;12:6425. doi: 10.1038/s41598-022-10178-z (PMC9018949; doi:10.1038/s41598-022-10178-z)
Supplement: Supplementary file 1 — Supplementary Information. [file 41598_2022_10178_MOESM1_ESM.pdf]

Structural equation modeling reveals decoupling of ecological and self-perceived outcomes in a garden box social-ecological system

Laura S. Tuominen[1,*], Samuli Helle[2], Heikki Helanterä[3], Patrik Karell[4], Lauri Rapeli[5], Douglas Richmond[1], Timo Vuorisalo[1], Jon E. Brommer[1]

University of Turku

[1]Department of Biology, University of Turku, Turku, 20014, Finland
[2]Department of Social Research, University of Turku, Turku, 20014, Finland
[3]Faculty of Science, University of Oulu, Oulu, 90014, Finland
[4]Bioeconomy Research Team, Novia University of Applied Sciences, Ekenäs, 10600, Finland
[5]The Social Science Research Institute, Åbo Akademi, Turku, 20500, Finland
[*]laura.s.tuominen@utu.fi

Supplementary Information

Supplementary Table 1.
List of the second-tier variables belonging to the SES framework and Evolutionary theory. Under "studied" we mark if the variables were included in this study or not. Under "Reasons/information" we either provide a brief argument for not studying the variables or a short description of how the variable is interpreted in our study.

variable name	studied	reasons / information
**Social, economic and political settings S**		
S1 economic development	no	no appreciable variation across different garden boxes within the study (")
S2 demographic trends	no	"
S3 political stability	no	"
S4 other governance systems	no	"
S5 markets	no	"
S6 media organizations	no	"
S7 technology	no	"
**Resource systems RS**		
RS1 sector	no	"
RS2 clarity of the system boundaries	no	"
RS3 size of resource system	yes	$1m^2$ x varying box number
RS4 human-constructed facilities	yes	different gardening effort
RS5 productivity of system	yes	shade
RS6 equilibrium properties	no	"
RS7 predictability of system dynamics	no	"
RS8 storage characteristics	no	"
RS9 location	yes	public / private
**Resource units RU**		
RU1 resource unit mobility	no	"
RU2 growth or replacement rate	no	"
RU3 interaction among resource units	no	"
RU4 economic value	no	perceived as the outcome of the activity
RU5 number of units	no	perceived as the outcome of the activity
RU6 distinctive characteristics	no	"
RU7 spatial and temporal distribution	no	"
RU8 social and other values	no	perceived as the outcome of the activity
**Governance systems GS**		
GS1 government organizations	no	"
GS2 nongovernment organizations	no	"
GS3.1 network structure	no	"
GS3.2 management strategy	no	"
GS4 property-rights system	no	"
GS5 operational-choice rules	no	"
GS6 collective-choice rules	yes	informal rules
GS7 constitutional-choice rules	no	"
GS8 monitoring and sanctioning processes	yes	if all the members were enough involved in work
**Actors A**		

A1.1 number of relevant actors	yes	gardening group size
A1.2 number of actors	yes	other gardeners in the same location
A2 socioeconomic attributes of users	no	not studied
A3 history or past experiences	yes	starting year, experienced damage and worries before summer
A4 location	no	”
A5 leadership / entrepreneurship	no	”
A6 norms (trust-reciprocity) /social capital	yes	social capital
A7 knowledge of SES/mental models	no	not studied
A8 importance of resource	no	”
A9 technologies available	no	”
**Evolutionary theory E**		
E1 repetition of interactions	yes	how often the group met
E2 relatedness	yes	the relatedness within the group
E3 stability of groups	yes	the stability of the group composition
**Interactions I**		
I1 harvesting levels of diverse users	no	perceived as the outcome of the activity
I2 information sharing among users	no	not studied
I3 deliberation processes	no	”
I4 conflicts	no	not studied
I5 investment activities	no	”
I6 lobbying activities	no	”
I7 self-organizing activities	no	”
I8 networking activities	no	”
I9 monitoring activities	no	”
I10 evaluative activities	no	”
**Outcomes O**		
O1 social performance measures	yes	self-perceived social, ecological or individual benefits
O2 ecological performance measures	yes	species number, quantity and quality of cultivations, economic value, area of weeds
O3 externalities to other SES	no	”
**Related ecosystems ECO**		
ECO1 climate patterns	no	”
ECO2 pollution patterns	no	”
ECO3 flows into and out of focal SES	no	”

Supplementary Table 2. *Survey Before the Summer 2019.*
There was a total of 107 answers to the questionnaire. The questionnaire is here presented in English, however the answers were mainly collected in Finnish (distributed also in Swedish). Compulsory questions marked with *.

Before summer garden box survey 2019

1. If you are involved in more than one garden box group, think about the group / locality you are mainly involved in when answering the questions.
   Are you registered as the contact person of your garden box group with the city of Turku? *

   ☐ Yes

   ☐ No

2. The name of your garden box / boxes *

____

3. When did you receive the first garden box from the city of Turku? *

   ☐ 2016

   ☐ 2017

   ☐ 2018

   ☐ 2019
4. How many boxes have you had each year?

   2016
   Total number of garden boxes _____

   2017
   Total number of garden boxes _____

   2018
   Total number of garden boxes _____

   2019
   Total number of garden boxes _____

5. How many people are in your gardening group? *

   ____

6. We are asking you to describe your relationship with the members of your group. Fill in a number for each given option describing how many of the people are primarily your family members, friends, neighbors and/or others.

   Family members (children and/or parents)
   Number ______
   Other family members
   Number ______
   Friends
   Number ______
   Neighbors

Number ______
Other
Number ______

7. What are you planning to cultivate in the garden boxes this summer? You can choose multiple options. *

☐ Flowers

☐ Vegetables

☐ Mushrooms

☐ Herbs

☐ Fruits

☐ Other, what ____

8. What expectations you have from the box gardening? Choose three most important options. *

☐ Fresh, quality produce
☐ Physical recreation
☐ Mental relaxation
☐ A way to beautify the area
☐ To educate my children
☐ Economic gain
☐ Aspiration towards self sufficiency
☐ To make new friends
☐ Sense of community
☐ To be a part of a social movement
☐ Quality time with friends / family
☐ Other reasons, what? ____________

9. How much money are you planning to use for the cultivations (€)? *

____

10. How much time are you planning to spend box gardening (hours / week)? *

____

11. The garden boxes are placed on public land, so are you concerned about the following factors damaging your cultivations? Please select to what extent you are concerned. *

	Not at all	Just a little	To some extent	Very concerned
Vandalism	○	○	○	○
Theft	○	○	○	○
Animals	○	○	○	○

12. Are you going to use some kind of method to prevent the damage? Choose maximum 3 options. *

☐ A net to cover the cultivations

☐ Observation

☐ Naming the boxes clearly

☐ Checking up the boxes regularly

☐ Scarecrows

☐ Other strategies, what ____

☐ No method

Supplementary Table 3. *Survey After the Summer 2019.*
There was a total of 79 answers to the questionnaire. It is here presented in English, however answers were mainly collected in Finnish (distributed also in Swedish). Compulsory questions marked with *.

After summer garden box survey 2019

1. If you are involved in more than one garden box group, think about the group you are mainly involved in when answering the questions
   Are you registered as the contact person of your garden box group with the city of Turku? *

   ☐ Yes

   ☐ No
2. The name of your garden box / boxes, or locality, if you can`t remember the name. *

____

3. The following question considers the size of your gardening box group and if stayed the same through summer 2019. With the gardening group we mean people who take care of the same shared boxes with you. How many people were in your gardening group including you? *
   Beginning of the summer ___________

   End of the summer ___________

4. Can you estimate how many people were box gardening with you in the same locality, if your boxes were near other people`s garden boxes? With the same locality, we mean about 20 meters distance from your boxes. *

   Number of people ___________

5. If you have been box gardening in previous years, has your garden box group members stayed the same?

   ☐ Yes, they have

   ☐ No, they haven`t

   ☐ Yes, partly
6. If you are planning to continue box gardening in the following years, do you think that your group is going to stay the same?

   ☐ Yes, they will

   ☐ No, they won`t

   ☐ Yes, partly
7. Please answer the following question if you have not answered to the first survey in the beginning of summer.
   We are asking you to describe your relationship with the members of your group. Fill in a number for each given option describing how many of the people are primarily your family members, friends, neighbors and/or others.

   Family members (children and/or parents)
   Number ______
   Other family members

Number ______
Friends
Number ______
Neighbors
Number ______
Other
Number ______

8. How often your group typically met during this summer? *

	every day	many times per week	once a week	few times per month	once a month	less than once a month
How often	◯	◯	◯	◯	◯	◯

9. Which of the following options best describes how your gardening group worked? You can choose several options. *

☐ I gardened alone my boxes

☐ Everyone in the group have their own separate boxes

☐ We agreed in advance on a plan how to take care of the shared boxes (e.g. watering)

☐ We were in contact with each other (e.g. WhatsApp) about taking care of the boxes

☐ We went together to take care of our cultivations

☐ Everyone went randomly to take care of the shared boxes

☐ Other ways, what? ____

10. Were all the members in your gardening group equally involved in taking care of the boxes? Mark the most suitable percentage to describe the whole summer`s involvement for each member. If there were, for example, two members in your gardening group and you took care of the boxes equally, mark for you and the 2. member 50 % and 50 %. If there were, for example, just two members in your group, mark rest “not a member”.

Estimation of the summer`s contribution (%)

	me	2. member	3. member	4. member	Rest of the members
not a member	☐	☐	☐	☐	☐
0	☐	☐	☐	☐	☐
10	☐	☐	☐	☐	☐
20	☐	☐	☐	☐	☐
30	☐	☐	☐	☐	☐
40	☐	☐	☐	☐	☐
50	☐	☐	☐	☐	☐
60	☐	☐	☐	☐	☐
70	☐	☐	☐	☐	☐

80	☐	☐	☐	☐	☐
90	☐	☐	☐	☐	☐
100	☐	☐	☐	☐	☐

11. If you felt that everyone in your gardening group did not take part equally in taking care of the cultivations, how did you deal with it? You can choose several options. *

☐ Everyone was involved enough

☐ They weren`t but I didn`t intervene

☐ We talked about it and members contributed more

☐ We talked about it, but it didn`t help

☐ Everyone didn`t get the same yield as they weren`t involved

☐ Other ways, what? ____

12. Did people not belonging in your official gardening group help you in taking care of the cultivations? We mean people who are gardening in the same locality or anyone who helped you (watering, weeding etc.).

☐ 1-2 people helped sometimes

☐ 1-2 people helped regularly

☐ many people helped sometimes

☐ many people helped regularly

☐ not at all

13. Did you help people who did not belong in your official gardening group? If you did, how often?

☐ I helped 1-2 people sometimes

☐ I helped 1-2 people regularly

☐ I helped many people sometimes

☐ I helped many people regularly

☐ not at all

14. Following questions consider encounters during the summer 2019. *

	Yes	No
Did you get to know to new people during summer through box gardening?	☐	☐
Did you meet other box gardeners during the summer?	☐	☐
Did you receive positive feedback for instance from passers-by?	☐	☐

Did you receive negative feedback for instance from passers-by?	☐	☐
Do you especially remember an encounter? How was it?	☐	☐

____

15. Do you feel that a community has formed around box gardening?

	Yes	No
I feel that box gardening has brought people together in the neighborhood	☐	☐
I feel that the gardener`s Facebook group is a community	☐	☐

16. Has box gardening created some other activities around it? Have people spent time together in other ways, such as organized a harvest party or something else? Tell us what?

____

17. Estimate the economic value of your cultivations (€) throughout the season. Try to estimate your cultivations so that you would have bought the same products from a shop during the summer. *

    ____

18. Did your cultivations succeed this summer? You can choose several options. *

    ☐ They succeeded perfectly

    ☐ Some of my cultivations were stolen

    ☐ Insects destroyed some cultivations

    ☐ Other animals ate/damaged some cultivations

    ☐ My cultivations dried

    ☐ My cultivations suffered from a plant disease

    ☐ Some other cause of damage for cultivations, what?

    ____

19. If your cultivations were stolen during the summer, did you change somehow your behavior? You can choose several options.

    ☐ No

    ☐ My cultivations were not stolen

    ☐ I increased the "surveillance" of my boxes

    ☐ I named better my boxes / put out a new sign

    ☐ I visited my boxes more often

    ☐ I asked other people to monitor my boxes

    ☐ I relocated my boxes

☐ I put a net to protect my cultivations

☐ Other, what?

20. If you have box gardened in previous years, has your cultivations been stolen / damaged before? *

☐ This was my first year

☐ Yes

☐ No

21. Reasons for gardening: possible benefits received from box gardening listed below. Choose for each benefit the option, which best describes your expectations of receiving this benefit before you started gardening this summer, and how much you received this benefits in the end. *

	I didn't expect, and didn't get	I did expect, but didn't get	I did expect, and did get a little	I did expect, and did get a lot	I didn't expect, but got a little	I didn't expect, but got a lot
Fresh, quality produce	○	○	○	○	○	○
Physical recreation	○	○	○	○	○	○
Mental relaxation	○	○	○	○	○	○
A way to beautify the area	○	○	○	○	○	○
To educate children	○	○	○	○	○	○
Economic gain and self sufficiency	○	○	○	○	○	○
New acquaintances	○	○	○	○	○	○
Sense of community	○	○	○	○	○	○
Quality time with friends / family	○	○	○	○	○	○
To increase my knowhow	○	○	○	○	○	○
Overall happiness	○	○	○	○	○	○
To be part of a social movement	○	○	○	○	○	○
To increase my nature connection	○	○	○	○	○	○
To increase the biodiversity in the area	○	○	○	○	○	○
Other reasons, what? ______	○	○	○	○	○	○

22. If you think you did not achieve something important that you expected from box gardening this summer, can you describe why?

---

23. Are you going to continue box gardening next summer? *

☐ Yes

☐ No

☐ I haven`t decided yet

24. If you continue box gardening next summer, are you going to do something different based on you experiences this summer? You can choose several options.

☐ I am not doing anything different

☐ I will cultivate different plants

☐ I will relocate the boxes, if possible

☐ I will put out a new sign

☐ I will have a different gardening group

☐ I will apply more boxes, if possible

☐ Other changes, what? ____

25. Please write you contact information, if you want to participate in possible interviews considering box gardening and in a lottery to win a movie ticket (Kino Diana).

First name ________________________________
Surname ________________________________
Phone number ________________________________
E-mail ________________________________

Supplementary Table 4. *Estimated Correlations for the Variables from Multiply Imputed Datasets.*
Estimated correlations between the measured variables belonging to the concepts Actors, Evolutionary theory (marked with *), Governance (marked with +) and Resource (marked with ^). The correlations over 0.400 are bolded (due to MI method, the p-values for correlations are not reported in Mplus).

**Variable**	starting year	worries	group size	damage	others number	social capital	stability*	related-ness*
starting year	1.000							
worries	-0.065	1.000						
group size	-0.172	0.033	1.000					
damage	-0.395	0.130	-0.025	1.000				
others number	0.046	0.131	**0.401**	-0.095	1.000			
social capital	0.018	-0.095	0.163	-0.093	0.154	1.000		
stability*	-0.172	-0.193	-0.164	0.088	-0.145	-0.080	1.000	
relatedness*	-0.060	-0.116	-0.056	0.102	-0.033	-0.033	0.024	1.000
group meetings*	0.206	0.155	-0.200	-0.157	-0.186	0.092	-0.057	0.049
involvement+	0.024	0.024	-0.098	-0.010	-0.226	-0.106	-0.095	0.023
rules+	-0.192	-0.183	0.315	-0.023	0.099	**0.448**	-0.024	-0.003
box number^	**-0.504**	0.162	0.167	**0.420**	-0.228	0.051	-0.008	0.006
effort^	-0.286	0.060	0.146	0.147	0.088	0.282	-0.006	-0.145
shade^	0.167	0.011	-0.259	-0.081	-0.129	-0.030	0.065	-0.238
privacy^	0.034	-0.028	0.173	-0.037	0.058	0.148	-0.027	-0.034

**Variable**	group meetings*	involvement+	rules+	box number^	effort^	shade^	privacy^
group meetings*	1.000						
involvement+	0.256	1.000					
rules+	-0.207	-0.070	1.000				
box number^	-0.012	-0.049	0.152	1.000			
effort^	0.110	0.058	0.213	0.283	1.000		
shade^	0.080	-0.114	-0.217	-0.017	-0.114	1.000	
privacy^	-0.119	0.107	0.135	0.043	0.083	-0.039	1.000

Supplementary Figure 1a. *Multiple imputation for the independent variables.*
There are variables included in the code, which are used in the data imputation to improve the quality of the results, but are not used in the analyses (moreb, org, org_alo, ysuc). The predictor variables are imputed (IMPUTE command) in the datasets and all the variables (USEVARIABLES command) are used in the imputation as they can be predictive of missingness (Asparouhov & Muthén 2010).

```
TITLE:          Multiple imputation for garden box data;
DATA:           FILE IS ansgpartdum.dat;
VARIABLE:       NAMES ARE
                    s_year moreb box_no gsize
                    related worries others_no stabil
                    gmeet org org_alo
                    rules involv
                    socicap ysuc damage weeds
                    effort eco_val privacy shade
                    qlty species cult_no cult_m2
                    receb ecob socib indib;
                USEVARIABLES ARE
                    s_year moreb box_no gsize
                    related worries others_no stabil
                    gmeet org org_alo
                    rules involv
                    socicap ysuc damage weeds
                    effort eco_val privacy shade
                    qlty species cult_no cult_m2
                    receb ecob socib indib;
                MISSING ARE ALL (-99);
DATA IMPUTATION:
                IMPUTE = s_year (c) box_no gsize related (c)
                    worries (c) other_no stabil (c) gmeet
                    org (c) org_alo (c)
                    rules (c) involv (c)
                    socicap damage (c)
                    effort (c) privacy (c) shade (c);
                 NDATASETS = 100;
                 SAVE = missimp*.dat;
ANALYSIS:        TYPE = BASIC;
```

Supplementary Figure 1b. *Social and Ecological Outcomes Model*

```
TITLE:              social and ecological outcomes;
DATA:               FILE IS ansgpartdum.dat;
VARIABLE:           NAMES ARE
                    s_year moreb box_no gsize
                    related worries others_no stabil
                    gmeet org org_alo
                    rules involv
                    socicap ysuc damage weeds
                    effort eco_val privacy shade
                    qlty species cult_no cult_m2
                    receb ecob socib indib;
                    USEVARIABLES ARE
                    ecob socib indib
                    weeds eco_val
                    qlty species cult_no cult_m2;
                    MISSING ARE ALL (-99);
DEFINE:             eco_val = eco_val / 10;
                    weeds = weeds / 10;
                    cult_no = cult_no / 100;
ANALYSIS:           ESTIMATOR = MLR;
                    ITERATIONS = 50;
MODEL:              soci_outcome BY socib@1 indib ecob;
                    soci_outcome;
                    eco_outcome BY species@1 eco_val
                    qlty weeds cult_no cult_m2;
                    eco_outcome;
OUTPUT:             STANDARDIZED TECH1 TECH4 MODINDICES(ALL 0)
```

Supplementary Figure 1c. *SES Framework and Evolutionary Concepts of Importance in Urban Garden Boxes*

```
OUTPUT:            STANDARDIZED TECH1 TECH4 MODINDICES(ALL 0)

TITLE:             SES-framework and Evolutionary concepts of importance in urban garden boxes;
DATA:              FILE IS missimplist.dat;
                   TYPE = IMPUTATION;
VARIABLE:          NAMES ARE
                   s_year moreb box_no gsize
                   related worries others_no stabil
                   gmeet org org_alo
                   rules involv
                   socicap ysuc damage weeds
                   effort eco_val privacy shade
                   qlty species cult_no cult_m2
                   receb ecob socib indib;
                   USEVARIABLES ARE
                   s_year box_no gsize
                   related worries others_no stabil
                   gmeet
                   rules involv
                   socicap damage weeds
                   effort eco_val privacy shade
                   qlty species cult_no cult_m2
                   ecob socib indib;
                   MISSING = *;
DEFINE:            eco_val = eco_val / 10;
                   weeds = weeds / 10;
                   cult_no = cult_no / 100;
ANALYSIS:          ESTIMATOR = MLR;
                   ITERATIONS = 50;
MODEL:             eco_out BY species@1 eco_val qlty weeds
                   cult_no cult_m2;
                   eco_out;
                   soci_out BY socib@1 indib ecob;
                   soci_out;
                   eco_out soci_out ON s_year box_no gsize
                   related worries others_no stabil
                   gmeet rules involv
                   socicap damage
                   effort privacy shade;
OUTPUT:            STANDARDIZED TECH1 TECH4 RESIDUAL
```

Supplementary Table 5. *Estimates for Ecological and Self-perceived Outcomes –Latent Variables and their Reflective Indicators.*

The table presents the latent variables and their reflective indicator factor loadings, standard errors for the loadings and p-values for the estimates. The unstandardized estimates are presented before the slash sign (/) and the standardized estimates after. The error terms for the reflective indicators signify the variance not explained by the respective latent variable they are loading onto.

**Latent variable**	**Reflective indicator**	**Factor loading**	**S.E.**	**p-value**	**Error term**	**S.E.**
**Self-perceived outcome BY**	social benefits	1.000 / 0.554	0.000 / 0.084	999.000 / < 0.001	2.698 / 0.693	0.440 / 0.093
	individual benefits	1.143 / 0.701	0.223 / 0.101	< 0.001	1.621 / 0.509	0.473 / 0.141
	ecological benefits	1.626 / 0.917	0.419 / 0.114	< 0.001	0.596 / 0.159	0.782 / 0.209
**Ecological outcome BY**	species diversity	1.000 / 0.815	0.000 / 0.079	999.000 / < 0.001	0.931 / 0.337	0.355 / 0.128
	economic value	0.764 / 0.665	0.172 / 0.068	< 0.001	1.356 / 0.558	0.559 / 0.091
	quality	0.209 / 0.391	0.063 / 0.084	0.001	0.444 / 0.847	0.065 / 0.066
	weeds	-0.171 / -0.317	0.055 / 0.051	0.002	0.481 / 0.899	0.173 / 0.032
	number of cultivations	0.253 / 0.454	0.057 / 0.071	< 0.001	0.452 / 0.794	0.166 / 0.064
	area of cultivations	0.107 / 0.635	0.025 / 0.091	< 0.001	0.031 / 0.597	0.008 / 0.115
